# Radiation‐Resistant Kr‐Selective MOF With Record Kr/Xe Selectivity at Elevated Pressure

**DOI:** 10.1002/advs.202600073

**Published:** 2026-05-04

**Authors:** Qihang Tian, Yinhui Li, Qingkuan Meng, Shizhen Liu, Yongzheng Wang, Bin Chen, Heping Ma

**Affiliations:** ^1^ State Key Laboratory of Fluorine & Nitrogen Chemicals School of Chemical Engineering and Technology Xi'an Jiaotong University Xi'an Shaanxi China

**Keywords:** gas adsorption and separation, metal organic framework, porous materials, radiation stability, xenon and krypton

## Abstract

The separation of Kr/Xe from spent nuclear fuel off‐gas is critical, yet most current adsorbents exhibit Xe‐Kr co‐adsorption under elevated pressures, posing challenges for industrial pressure swing adsorption processes. Herein, we present and evaluate a metal‐organic framework, CALF‐20M‐w, engineered for molecular sieving separation of Kr/Xe under industrial pressure conditions. CALF‐20M‐w possesses a pore size of 3.7 Å, which position between Kr (3.6 Å) and Xe (4.1 Å) kinetic diameters, enables record Kr/Xe uptake ratio (52.2) and selectivity of 4424 at 195 K and 5 bar, surpassing all benchmark materials. GCMC simulations reveal that Kr atoms occupy favorable positions within CALF‐20M‐w's intersecting channels, while Xe atoms are sterically excluded in the pressure range of 1–30 bar. Dynamic breakthrough separation experiments and PSA simulation show CALF‐20M‐w can yield >99.9% pure Xe, demonstrating feasibility for radioactive Kr removal from spent nuclear fuel off‐gases. Moreover, CALF‐20M‐w exhibits extreme radiation resistance under β‐irradiation (72 kGy/h) and γ‐irradiation (240 kGy), outperforming UiO‐66 and ZIF‐8. CALF‐20M‐w sets new benchmarks for high‐pressure molecular sieving in Kr/Xe separation, offering a highly promising strategy for nuclear waste management and rare gas purification.

## Graphical abstract

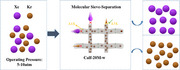

## 1 Introduction

**SCHEME 1:**
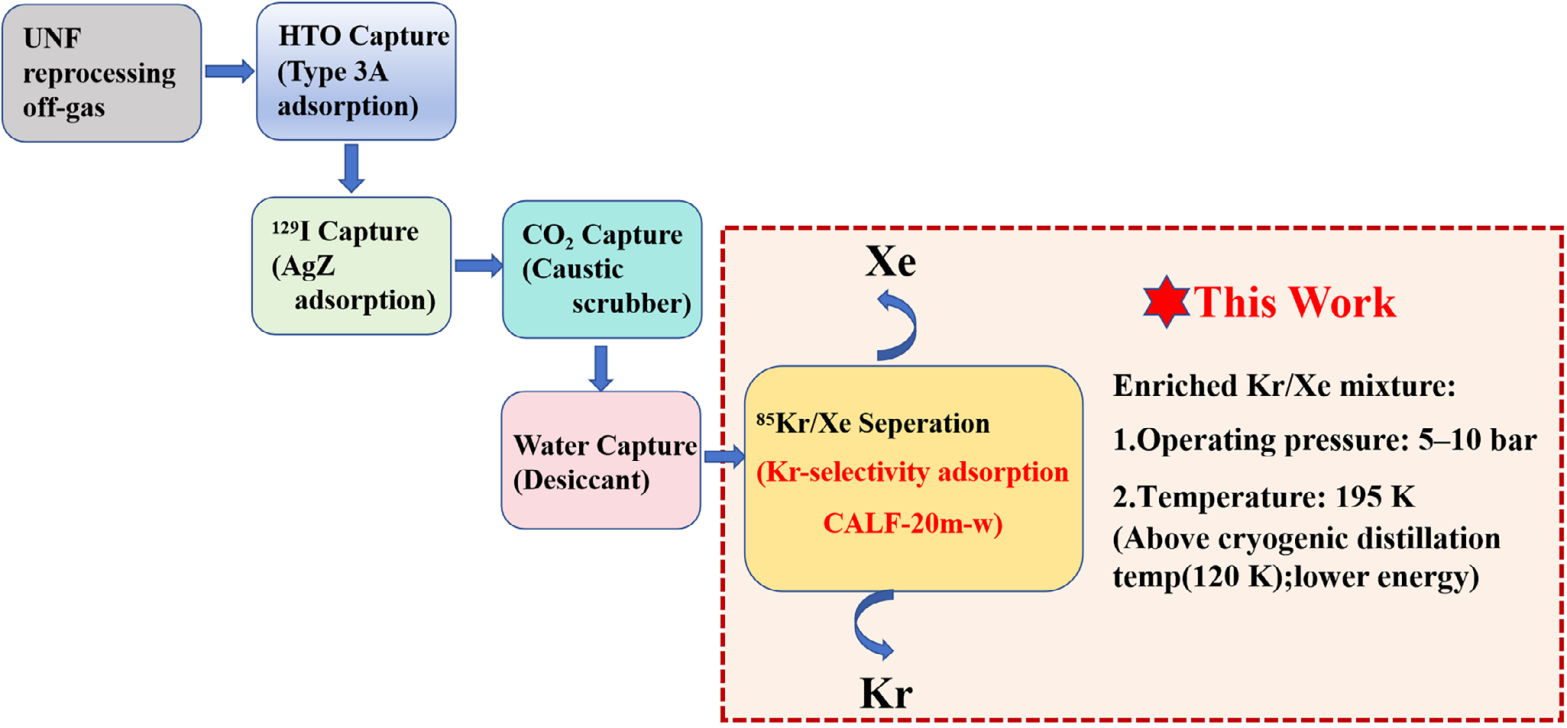
Schematic of UNF reprocessing off‐gas system.

In recent years, nuclear power has emerged as a reliable, cost‐effective, and clean energy source, owing to its high energy density, low CO_2_ emissions, and minimal land footprint. Nevertheless, its broader deployment is hindered by the challenges of managing radioactive waste produced during the reprocessing of spent nuclear fuel (SNF) [1]. As depicted in Scheme 1, due to their high chemical reactivity, ^3^H and ^129^I are typically captured using 3A molecular sieves and silver‐exchanged zeolites. In the case of irradiated nuclear fuel, most of the ^14^C is released in the form of ^14^CO_2_, which is presently managed by a caustic liquid scrubbing system [2]. Currently, the recovery and separation of ^85^Kr and stable xenon isotopes rely on cryogenic distillation—a highly energy‑intensive process typically operated at around 120 K, associated with significant operational expenses. Furthermore, this distillation step carries a safety risk due to the accumulation of ozone, which may lead to explosive conditions. In contrast, an alternative approach based on physisorption technology offers an energy‑efficient, cost‑effective, and environmentally benign means for the selective separation of complex gas mixtures [3]. This work intends to separate the high‐pressure mixed gas (5–10 bar) after Kr/Xe enrichment at 195 K (significantly higher than the ∼120 K of cryogenic distillation, with lower energy consumption).

Xenon (Xe) and krypton (Kr) isotope generated from nuclear fission reactions serve as critical signature gases in spent nuclear fuel (SNF) process since its high fission yields. The Xe and Kr concentrations in SNF off‐gases is dozens to hundreds of times higher than in the atmosphere, giving it significant recovery value [4]. Xe is commonly used in lighting, electronic chips, anesthetics and propellants for ion propulsion engines. Radioactive ^85^Kr can be used as gas radiation sources for thickness gauges, densitometers, electron capture testers, aerosol neutralizers and so on. Therefore, separating Kr and Xe in SNF off‐gases has significant commercial value [5, 6].

The task of capturing and separating Xe and Kr from SNF off‐gases is currently adopting adsorption separation technology [7, 8]. Zeolites, porous carbons and metal‐organic framework (MOFs) have been widely studied for the separation of Xe/Kr [9, 10]. Since Xe has larger molecular size and higher polarizability than Kr [11], existing porous adsorbents tend to preferentially adsorb Xe over Kr. While in SNF off‐gases, the radioisotope ^85^Kr has a longer half‐life (10.8 years) than ^127^Xe (36.3 days), making removal of Kr is higher priority [12]. The Kr‐selective adsorbents have the ability to capture Kr and produce pure Xe directly, which is more suitable for Kr/Xe separation in SNF off‐gases. However, only a few Kr‐selective adsorbents have been reported so far [13, 14]. Thallapally et al. reported partially fluorinated metal organic frameworks (FMOFCu) with different cavity size that showed an inversion in sorption selectivity toward Kr at temperatures below 0°C. The 1D microtubes in FMOFCu appear Kr/Xe selectivity of 36 at 0.1 bar and 203 K [15]. Li et al. reported a flexible metal organic framework with adsorbate‐ and temperature‐dependent adsorption behavior toward Xe‐Kr gases. The guest and temperature dependence of the structural breathing in flexible MOF gives rise to a reverse of Xe/Kr adsorption selectivity, which showed Kr‐selective adsorption at 200K [14]. Bao et al. reported Kr‐selective carbon molecular sieve named C‐Suc‐750, which can achieve Kr/Xe sieving with a remarkable Kr/Xe uptake ratio of 39.3 at ambient conditions, setting a new benchmark for selective Kr adsorption [16]. Hong and Lee et al. reported a Hofmann‐type framework (CoNi‐DAB) showed high Kr/Xe (0.8/0.2) adsorption selectivity of Kr/Xe uptake ratio of 7 at 195K [13]. Elsaidi et al. presents a transformation of the CuBTC, into an Kr‐selective adsorbent by systematic compression of MOF nanoparticles to densified CuBTC phase, which exhibits selective adsorption for Kr over Xe with Kr/Xe selectivity of 3.8 [17].

Another critical issue is that current research on Xe‐Kr separation adsorbents is predominantly conducted at ambient pressure, which significantly deviates from the actual operating pressures (typically 5–10 atm) of industrial pressure swing adsorption (PSA) processes [18, 19]. Under such elevated pressures, most porous adsorbents exhibit priority adsorption of Xe and Xe‐Kr co‐adsorption, leading to the reduce of Xe purity and recovery rates. Consequently, evaluating adsorbent performance for Xe/Kr separation at higher pressures is more relevant for practical industrial applications. Theoretically, adsorbents with molecular sieving capabilities can enhance gas processing efficiency while maintaining separation performance under elevated pressures, rendering them more suitable for industrial PSA applications [17]. The monatomic nature of Xe and Kr gases results in spherical molecular structures that exhibit high symmetry in 3D space, making Kr/Xe molecular sieving separation is more difficulty [4]. To date, no adsorbent other than C‐Suc‐750 has achieved molecular sieving separation of Kr/Xe at 1 bar [16]. In this study, we synthesized a Zn‐methyl triazole‐based MOF, called CALF‐20M‐w [Zn (Mtz) (ox)_0.5_] for Kr/Xe molecular sieving separation at 5 bar pressure, which is approaching the actual operating pressure in PSA [19]. CALF‐20M‐w has cross‐interacting 2D channels, with an accessible pore‐throat of 3.7 Å, which is just between the kinetic diameters of Kr (3.6 Å) and Xe (4.1 Å). Computational simulations (GCMC) reveal the constricted throats of CALF‐20M‐w channels showed excellent Kr sieving effect over Xe. At 195 K and 5 bar, the IAST selectivity of Kr/Xe in CALF‐20M‐w reaches 4424, which exceeds all current reported Kr/Xe separation adsorbents. The adsorption capacity ratio of Kr/Xe = 0.8/0.2 in CALF‐20M‐w was as high as 52.2, which is also the highest value reported so far. The irradiation stability of CALF‐20M‐w after β and γ irradiation was monitored by powder X‐ray diffraction (PXRD) and adsorption isotherms. Using the total photon cross section, coordination number and crystal orbital overlapping population (COOP) calculations for different elements, the mechanism of the excellent irradiation stability of CALF‐20M‐w was investigated in detail. Moreover, fixed‐bed breakthrough experiments were performed under dynamic conditions to evaluate the potential of CALF‐20M‐w for practical adsorptive separation of Kr over Xe from SNF off‐gases.

## 2 Results and Discussion

**FIGURE 1:**
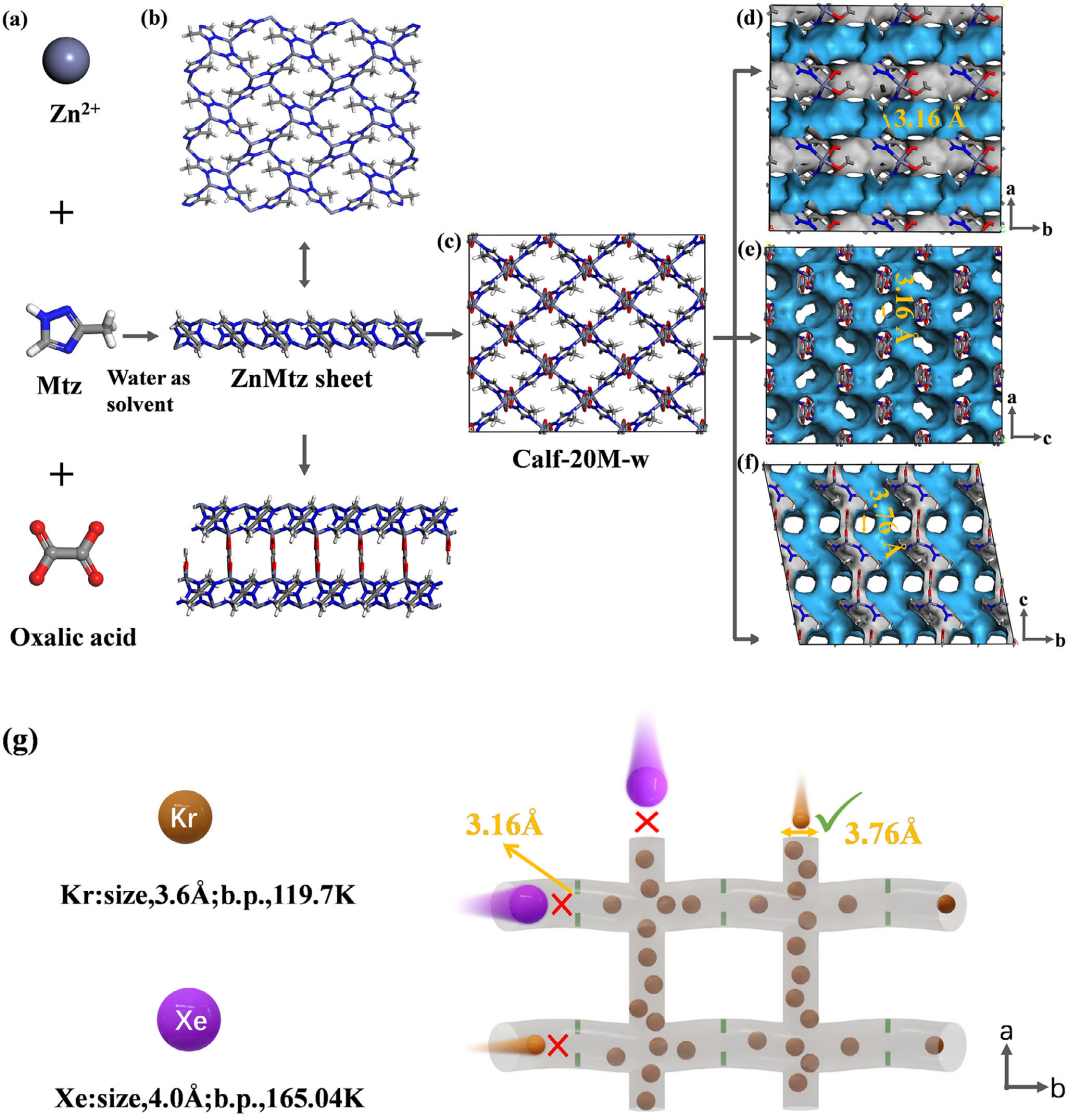
(a, b) Synthesis route of CALF‐20M‐w, schematic illustrations of the 2D zinc triazolate layers bearing methyl functional groups, and their linkage via oxalic acid pillars; (c) 3D pillared‐layer framework of CALF‐20M‐w viewed along the b‐axis; (d–f) Connolly surface representation viewed along the c‐axis, b‐axis and a‐axis; (pore surfaces depicted in light blue). Color scheme: carbon, gray; nitrogen, blue; oxygen, red; zinc, cyan; hydrogen, white; (g) A 3D schematic model depicting the molecular sieving mechanism of Kr/Xe separation through the pore channels of CALF‐20M‐w.

CALF‐20M‐w crystals was prepared by hydrothermal synthesis following previous literature [20]. The powder X‐ray diffraction patterns verified the good phase purity of the mass‐synthesized product (Figure S1). CALF‐20M‐w has five coordinated Zn ions in a distorted triangular biconical geometry, each Zn ion being pentacoordinated with three N atoms of three independent 3‐methyl‐1H‐1,2,4‐triazole (Mtz) ligands and two O atoms of one oxalate (Ox) (Figure 1a,b; Figure S2). The formed 2D undulating layer was further supported by oxalate columns to generate a 3D framework with 2D penetrating pores (Figure 1c). The intersecting channels in CALF‐20M‐w exhibits a periodically restricted pore throat due to the spatial hindrance effect of methyl groups. As shown in Figure 1d,e, CALF‐20M‐w has intersecting channels but a restriction throat of about 3.1 Å along b axis (Figure 1d). These narrow pore throats are significantly smaller than the kinetic diameters of Xe (4.1 Å) and Kr (3.6 Å), which no longer possess sieving properties (Figure 1e). The accumulation between adjacent layers is supported by oxalic acid, forming a continuous 1D pore with a pore diameter of t 3.7 Å. This pore size is slightly larger than the kinetic diameter of Kr (3.6 Å) but smaller than the kinetic diameter of Xe (4.1 Å). This 3.7 Å pore in CALF‐20M‐w can serves as a precise sieving of Kr/Xe. At the same time, the incoming Kr can diffuse locally in the pore channel with a limiting throat of 3.1 Å, which also determines that CALF‐20M‐w has a considerable adsorption capacity (Figure 1f).

The 77 K N_2_ adsorption of CALF‐20M‐w indicated no detectable N_2_ uptake for the activated material (Figure S3). To evaluate the specific surface area of CALF‐20M‐w, CO_2_ was employed as a probe (Figure S4). The BET specific surface area of CALF‐20M‐w was 241.86 m^2^·g^−1^ calculated from the CO_2_ adsorption isotherm at 273 K. Meanwhile, the BET surface area of CALF‐20M‐w calculated from the Ar adsorption isotherm at 87 K is 236.4 m^2^·g^−^^1^(Figure S5). Scanning electron microscopy (SEM) images showed that CALF‐20M‐w presented irregular crystals with diameters of 5–20 µm (Figure S6a). The energy dispersive X‐ray (EDX) facet scans show that C, N, O and Zn are uniformly dispersed in CALF‐20M‐w (Figure S6b‐e). The atomic ratios of C, N, O, and Zn obtained from EDX analysis are 43.24%, 31.13%, 15.15%, and 10.47%, respectively (Table S2), which are in good agreement with the theoretical values (C_4_H_4_N_3_O_2_Zn, C:40%, N:30%, O:20%, and Zn:10%).

**FIGURE 2:**
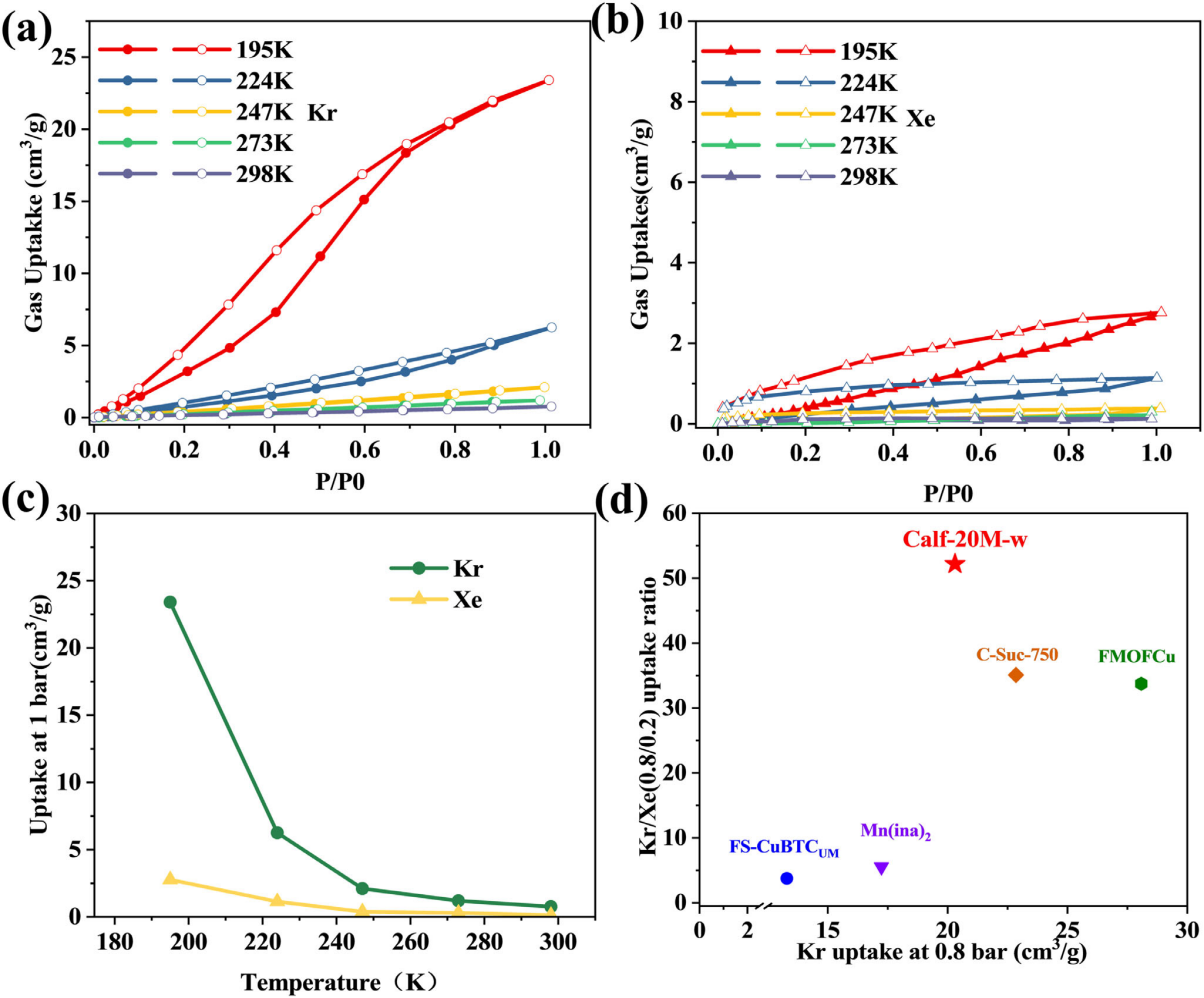
(a, b) Adsorption isotherms on CALF‐20M‐w for different temperatures Kr and Xe at atmospheric pressure. (c) Adsorption of Kr and Xe as a function of temperature at 1 bar. (d) Plot of Kr uptake at 0.8 bar versus the ratio of Kr/Xe uptake at 0.8/0.2 bar at 195 K for CALF‐20M‐w and other benchmark materials for separated Kr/Xe.

Adsorption/desorption isotherms of pure Kr and Xe on CALF‐20M‐w were conducted at five distinct temperatures. As shown in Figure 2a,b, the Kr uptake in CALF‐20M‐w at 298 and 273 K under 1 bar was 0.7 and 1.19 cm^3^·g^−^^1^, respectively; while Xe adsorption was 0.3 and 1.1 cm^3^·g^−^^1^. Upon reduction of the testing temperature, the adsorption capacities of Kr in CALF‐20M‐w increased to 23.06 cm^3^·g^−^^1^, which is far exceeded that of Xe (2.76 cm^3^·g^−^^1^) (Figure 2c). Moreover, the adsorption of Kr in CALF‐20M‐w at 195K showed a gently stepped profile similar to the one usually observed for flexible MOFs [21]: it increased linearly until 0.4 bar, and there was a steep increasing trend of adsorption between 0.4 and 0.7 bar, and then continues to increase in a linear trend after 0.7 bar and still does not reach a plateau at 1 bar. At 195K and 0.8 bar(the reference pressure for evaluating the adsorbent's capability to capture Kr from industrial Xe/Kr (0.2/0.8) mixtures), the corresponding Kr/Xe adsorption ratio in CALF‐20M‐w reached 52.2, surpassing all reported Kr‐selective adsorbent materials such as C‐Suc‐750 (35.09) [16], FS‐CuBTC_UM_ (3.75) [17], FMOFCu (33.73) [15] and Mn(ina)_2_ (5.53) [14] (Figure 2d). It is worth mentioning that CALF‐20M‐w exhibited a linear Xe adsorption isotherm, which may be attributed to trace surface adsorption on the material (Figure 2b). The Kr/Xe selectivity of the binary mixture was calculated using the Ideal Adsorption Solution Theory (IAST). As shown in Figure S7, the Kr/Xe selectivity of CALF‐20M‐w is about 15 under initial pressure.

**FIGURE 3:**
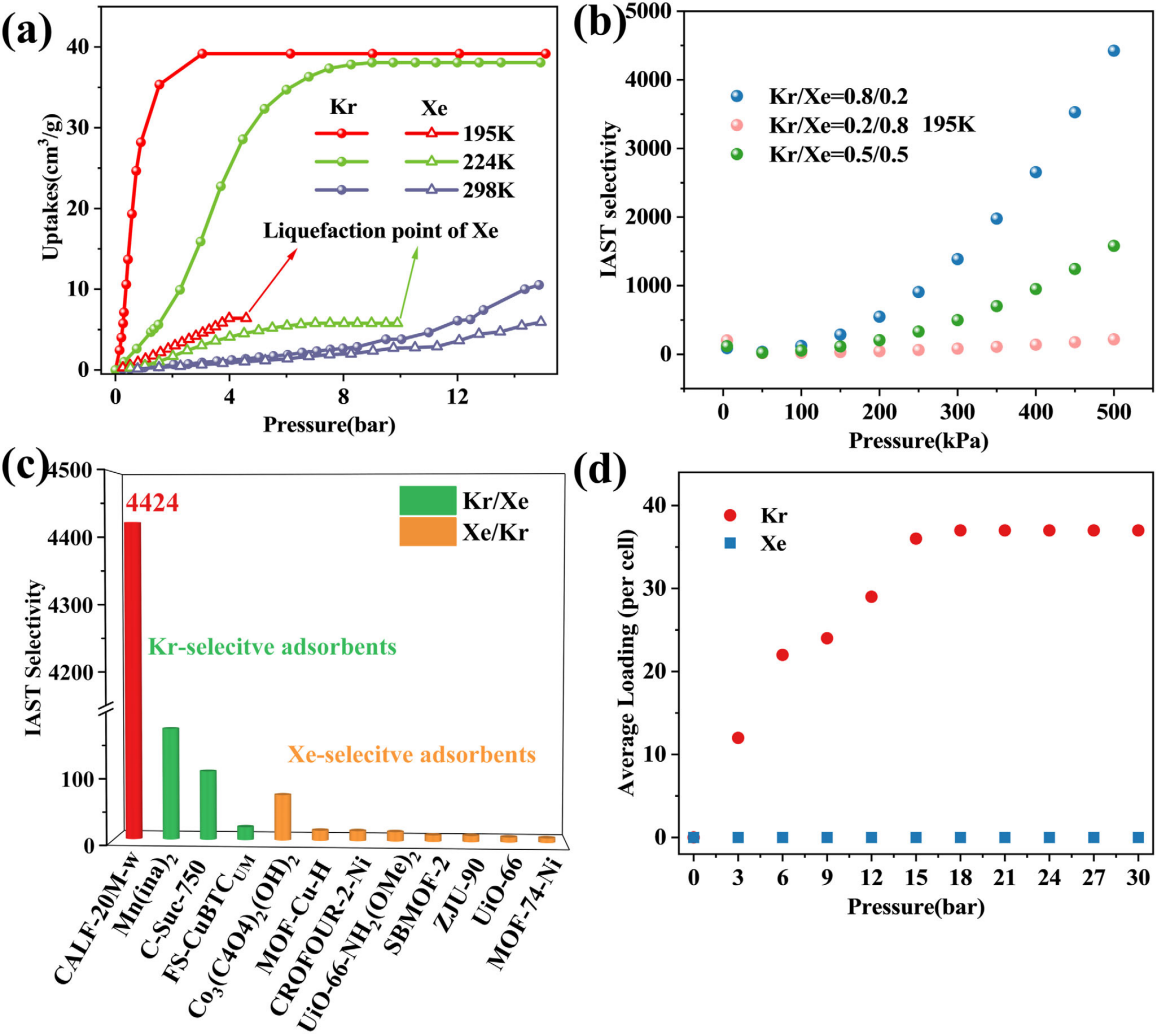
(a) Adsorption isotherms of Kr and Xe by CALF‐20M‐w at different temperatures and higher pressures (b) IAST selectivity of Kr/Xe at (0.8/0.2, 0.5/0.5, 0.2/0.8; v/v) at 195 K and 5 bar, respectively. (c) Comparison of the IAST selectivity of CALF‐20M‐w with reported adsorbents for Xe/Kr or Kr/Xe at 5 bar pressure. (d) Average loading of Kr and Xe in per cell of CALF‐20M‐w.

Since the operating pressures of industrial PSA processes is typically at 5–10 bar, we further investigated the Kr and Xe adsorption of CALF‐20M‐w under higher pressures (Figure 3a). At 195 and 224 K, the Kr adsorption capacity rapidly increase along with the rising of pressure. Under 195 K and 5 bar (close to Xe's liquefaction point pressure), the Kr saturation capacity reached 39.17 cm^3^/g, whereas Xe adsorption was only increased to 6.4 cm^3^/g. Similarly, at 224 K and 10 bar (close to Xe's liquefaction point pressure), the saturation capacities for Kr and Xe were 38.07 cm^3^/g and 5.8 cm^3^/g, respectively. The marked disparity in saturation capacities demonstrates CALF‐20M‐w can achieve Kr‐selective adsorption under high‐pressure conditions. Based on the single‐component equilibrium gas adsorption isotherm, the IAST selectivity of CALF‐20M‐w for Kr/Xe mixtures at 5 bar was calculated (Figure 3b; Figure S8). Notably, the IAST selectivity for Kr/Xe (0.2/0.8, v/v), Kr/Xe (0.5/0.5, v/v), and Kr/Xe (0.8/0.2, v/v) at 195 K and 5 bar reached 217, 1580, and 4424, respectively, representing the highest reported IAST selectivity to date. These values far surpass the IAST selectivity of benchmark materials such as C‐Suc‐750 [16] (105), FS‐CuBTC_UM_ [17] (20), conclusively demonstrating CALF‐20M‐w's preferential adsorption of Kr over Xe under elevated pressure conditions (Figure 3c), highlighting its molecular sieving separation of Kr/Xe. To further understand the adsorption difference of Kr and Xe within CALF‐20M‐w, we performed theoretical calculations using giant canonical Monte Carlo (GCMC) simulations (see Supporting Information for detail). GCMC simulations show that Kr molecules occupy favorable positions in CALF‐20M‐w pores, whereas Xe molecules are almost inaccessible to the pores due to the size limitation, and only a small amount of Xe adsorbed on the surface (Figure S9). In addition, the average loading of Kr and Xe in CALF‐20M‐w cells at different pressures indicates that Kr atoms can enter the pores when the pressure is increased to 3 bar, while Xe atoms could hardly enter the pores even when the pressure was increased to 30 bar. This result further proves that CALF‐20M‐w can realize the precise sieving separation of Kr/Xe (Figure 3d).

**FIGURE 4:**
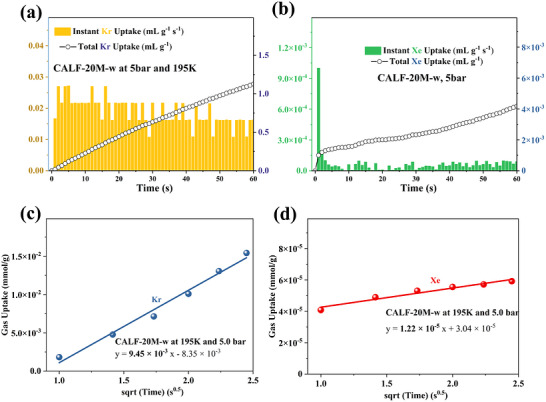
(a–d) The adsorption kinetics and diffusion coefficients of Kr and Xe on CALF‐20M‐w at 195 K and 5 bar.

To investigate the adsorption kinetic of Xe and Kr in CALF‐20M‐w, we measured the Xe/Kr adsorption rates in CALF‐20M‐w at 195 K and 5 bar. As shown in below and Figure 4, the Kr adsorption rate in CALF‐20M‐w is 0.02 mL g^−1^s^−1^, and the Kr uptake per unit time remains basically unchanged throughout the entire testing period, proving that the diffusion of Kr inside the CALF‐20M‐w remains stable (Figure 4a). Whereas for adsorption rates of Xe in CALF‐20M‐w, its maximum adsorption rate is only 0.00095 mL g^−1^s^−1^ at initial stage, and then immediately decreases to 0.00006 mL g^−1^s^−1^, proving that Xe is basically not adsorbed in CALF‐20M‐w (Figure 4b). Based on adsorption kinetic of Xe and Kr in CALF‐20M‐w, we performed linear fitting on the adsorption capacity and time data to calculate the diffusion coefficients of Xe and Kr within CALF‐20M‐w, using their ratio as the kinetic selectivity (Figure 4c,d). The results indicate that CALF‐20M‐w exhibit high Kr/Xe kinetic selectivity of 600000, which further corroborates the kinetic Kr/Xe molecular size‐sieving separation mechanism in CALF‐20M‐w.

**FIGURE 5:**
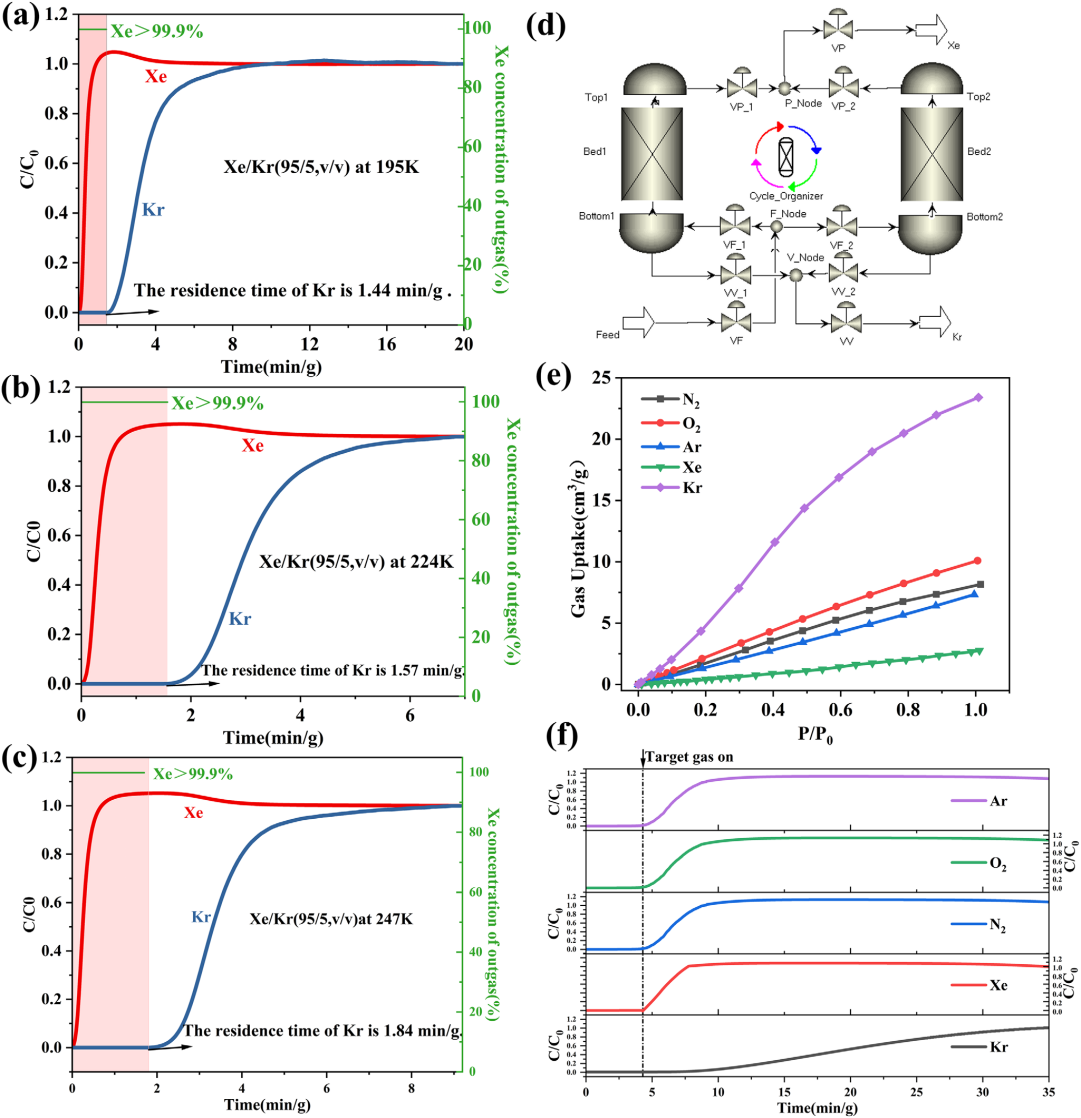
Breakthrough experiments. (a) 195 K (b) 224 K (c) 247 K Column breakthrough experiments under Kr/Xe (0.05/0.95, v/v) with CALF‐20M‐w. (d) Schematic model for 2‐bed PSA process. (e) Adsorption isotherms of Xe, Kr, N_2_, O_2_, and Ar on CALF‐20M‐w at 195 K. (f) Simulated nuclear waste reprocessing gas on CALF‐20M‐w at 195 K and 5 bar.

Building upon the exceptional Kr/Xe molecular sieve performance demonstrated above, we conduct Kr‐Xe binary breakthrough experiments under 247, 224, 195 K (Figure S14). Dynamic breakthrough experiments show the CALF‐20M‐w adsorption bed can achieve high‐purity Xe (>99.9%) at all three temperatures (247, 224, and 195 K). As shown in Figure 5a‐c, Xe exhibits sharp breakthrough profiles at all temperatures, indicating immediate elution of Xe with negligible retention in the column. In contrast, Kr displays a retention time of 1.84 min/g in the column under these conditions. The stark disparity in breakthrough times between Kr and Xe validates the exceptional molecular sieving capability of this adsorbent. We analyzed the similarity of the breakthrough curves observed at different temperatures: In the breakthrough experiments, since the residence time of the mixed gas in the column is relatively short, Kr do not have sufficient time to diffuse to all internal pores reaching adsorption saturation. As the temperature decreases, the residence time of Kr in the breakthrough column shortens from 1.84 min/g to 1.44 min/g, which can be attributed to the decrease of Kr diffusion rate in the CALF‐20M‐w at lower temperature (Figure 5a‐c). Further variation of the Xe/Kr ratio to 0.2/0.8 and 0.9/0.1 at 195 K (Figure S10) revealed that Xe consistently exhibits immediate breakthrough, whereas Kr maintains a protracted breakthrough trend with reduced retention time. These observations align with the Kr/Xe selectivity predicted by Ideal Adsorption Solution Theory calculations, further corroborating the consistency between experimental and theoretical analysis of Kr/Xe molecular sieving separation in CALF‐20M‐w.

The practical capability of CALF‐20M‐w for Kr/Xe separation was evaluated using a 2‐bed pressure swing adsorption (PSA) simulation process (see Section S1.7 for details). The results demonstrate that, under feed conditions of Kr/Xe (20/80), CALF‐20M‐w achieves a Xe purity of 99% (Figure 5d; Table S5). This indicates the material's significant potential for large‐scale industrial applications. To evaluate the potential of CALF‐20M‐w for Kr extraction from off‐gases in nuclear waste reprocessing, single‐component adsorption isotherms of Xe, Kr, N_2_, O_2_, and Ar were collected at different temperatures. At 195 K and 1.0 bar, the equilibrium uptake of Xe, N_2_, O_2_, and Ar on CALF‐20M‐w was measured to be 2.76, 8.16, 10.08, and 7.32 cm^3^/g, respectively, which was significantly lower than the Kr uptake of 23.4 cm^3^/g (Figure 5e). The Kr capture capability of CALF‐20M‐w under industrial conditions were evaluated using Aspen ADSIM for a simulated nuclear reprocessing off‐gas mixture (400 ppm Xe, 40 ppm Kr, balanced with air) at an elevated pressure of 5 atm (Figure 5f; Section S1.7). The results reveal that weakly adsorbed Ar, N_2_, and O_2_ were immediately eluted, while the larger Xe molecules were eluted even more rapidly due to size exclusion. In contrast, Kr was successfully separated and only began to slowly breakthrough the column at 3 min/g, highlighting the unique capability of this material to selectively adsorb Kr from a mixture of inert gases.

**FIGURE 6:**
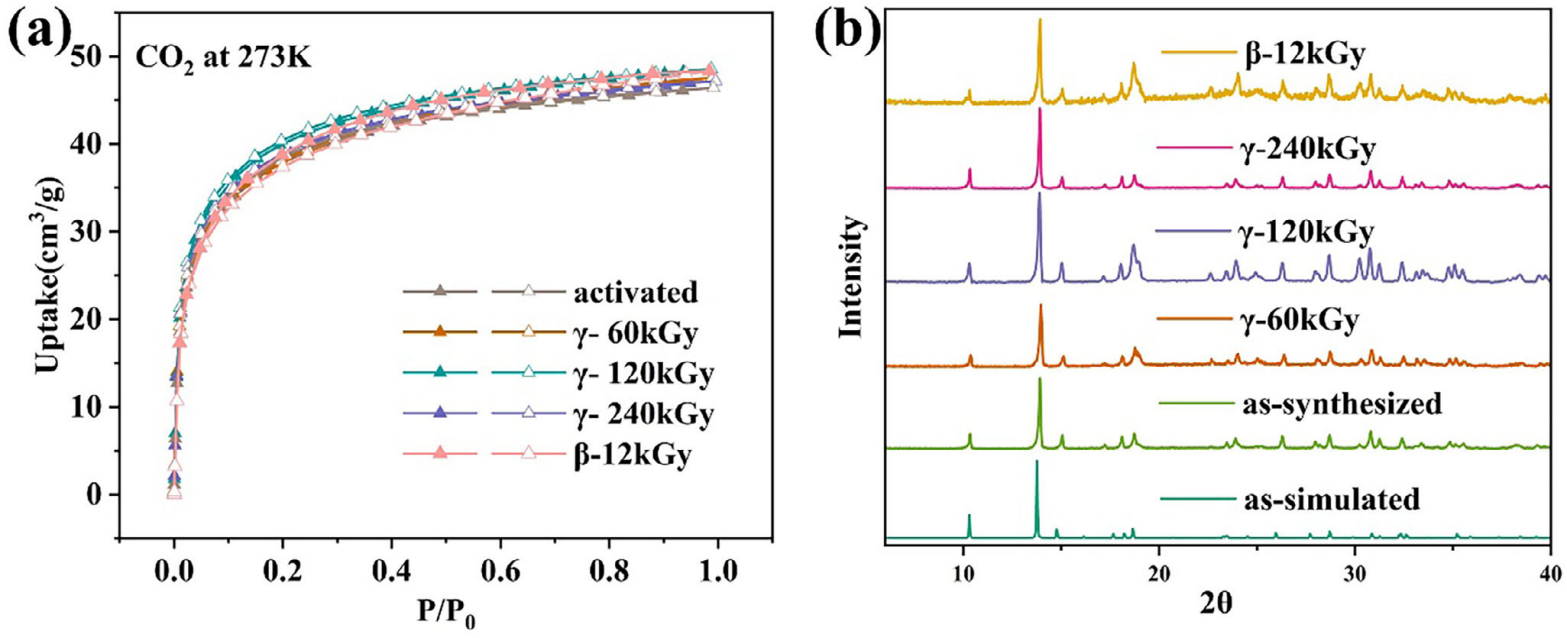
(a) 273K CO_2_ adsorption isotherms of CALF‐20M‐w samples under β/γ irradiations. (b) Powder X‐ray diffractograms of CALF‐20M‐w samples under β/γ irradiations.

The long‐term viability of adsorbents for nuclear industry applications depends critically on their radiation stability [22, 23, 24]. The decay modes of ^85^Kr and ^127^Xe are mainly β‐decay and a small amount of γ‐decay [25]. Consequently, we evaluated the radiation stability of CALF‐20M‐w under the both β and γ exposure conditions: 1) β‐electron beam irradiation dose rate was approximately 72 kGy/h for the beam flux density and energy of 5 × 10^−^^4^ A/m^2^ and 20 keV, see Figure S11 and Supporting Information for details; and 2) γ‐ray irradiation with total doses ranging from 60 to 240 kGy. First, CO_2_ adsorption isotherm at 273 K is used to monitor β‐irradiated samples (β‐CALF‐20M‐w), which demonstrated nearly equivalent CO_2_ uptake compared to non‐irradiated counterparts (Figure 6b). Second, after γ‐ray irradiation at 60 kGy, 120 kGy, and 240 kGy, CO_2_ adsorption isotherms measured under identical conditions (273 K, 1 bar) showed no detectable reduction in adsorption capacity in all γ‐irradiated samples (Figure 6b). These radiation dosage is significantly higher than the reported values of MIP‐203‐F‐Br (100 kGy) [26], SIFSIX‐3‐Cu (50 kGy) [27], Al‐Fum (10 kGy) [28], Al‐Fum‐Me (10 kGy) [28], Zr‐Fum‐Me (8 k Gy) [29], SIFSIX‐3‐Co (3 kGy) [27], and UiO‐66 (Zr) (2 kGy) [30] adsorbents, which indicate excellent radioactivity stability of CALF‐20M‐w. In addition, the structural changes of β‐CALF‐20M‐w and γ‐CALF‐20M‐w (irradiated with cobalt‐60 at 60–240 kGy) were monitored by PXRD as shown in Figure 6a. The PXRD patterns indicate that β‐CALF‐20M‐w and γ‐CALF‐20M‐w still maintain their crystallinity. Finally, the morphology of CALF‐20M‐w after irradiation treatment was observed by scanning electron microscopy (Figure S12), and it was found that the morphology was almost unchanged compared with the original sample, which again illustrated the radiation stability of CALF‐20M‐w.

**FIGURE 7:**
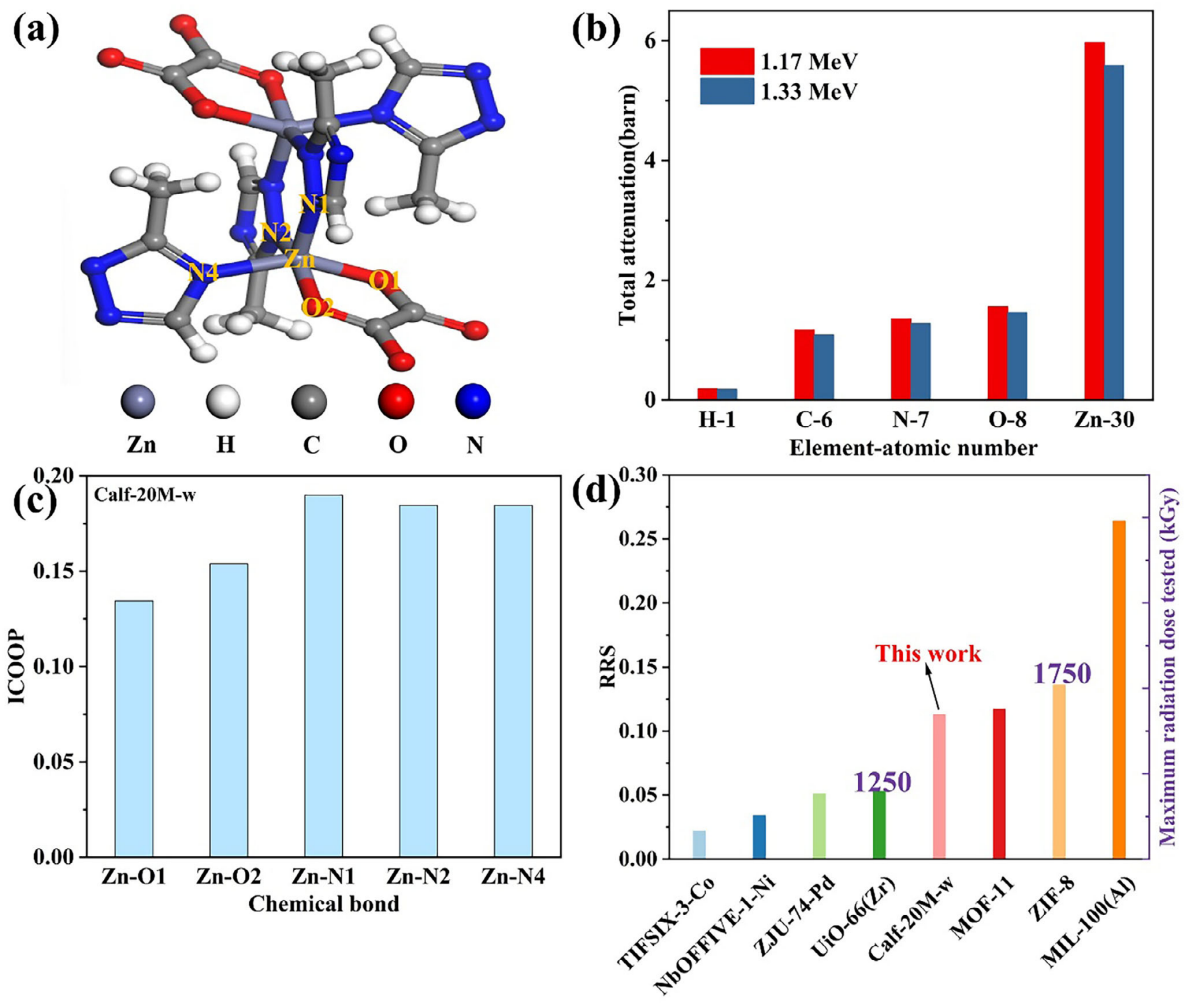
(a) Metal‐to‐pillar complexes of CALF‐20M‐w. (b) Total photon cross sections of different elements, 1 barn = 10^−24^ cm^2^. (c) COOP curves and (d) ICOOP curves for CALF‐20M‐w. (d) Relative radiation stability for CALF‐20M‐w as compared to benchmark radiation stable MOFs. (Color scheme: carbon, gray; nitrogen, blue; oxygen, red; zinc, cyan; hydrogen, white).

The relative radiation stability was used to further understand the radiation stability of CALF‐20M‐w [25]. The cross section of ^60^Co energy of CALF‐20M‐w element used in this study is shown in Figure 7b. The photon cross section of different atoms measures the average fraction of photon energy absorbed by the material, which can be used as an effective indicator to assess the radiative stability. Second, the chemical bond strength is complementary to the evaluation of the radiative stability of the material, and the concept of the crystal orbital overlapping population (COOP) number, calculated from the projection of the density of states onto a particular molecular bond, is a complementary tool for the study of the strength of the chemical bond. The photon cross sections of metal atoms in the CALF‐20M‐w are determined as Zn (5.97 barn at 1.17 MeV and 5.59 barn at 1.33 MeV), as illustrated in Figure 7b. Due to the distinct coordination environments of Zn‐O and Zn‐N in CALF‐20M‐w, it is essential to differentiate them in Crystal Orbital Overlap Population (COOP) calculations (Figure 7a). The calculated Integrated COOP (ICOOP) values for Zn‐O1, Zn‐O2, Zn‐N1, Zn‐N2, and Zn‐N3 are 0.13443, 0.15378, 0.18989, 0.18456, and 0.18456, respectively (Figure 7c). This indicates that multiple chemical bonds between Zn and the pillar ligands maintain relative stability under γ‐radiation. To reflect the material's intrinsic stability, the Zn─O1 bond with the lowest bond energy was selected for subsequent Radiation Resistance Stability (RRS) calculation. Finally, the RRS results of CALF‐20M‐w were obtained by combining the metal coordination numbers, which were compared with the RRS of radiation‐stable MOFs reported in the literature (irradiation intensities of MOFs reported in the literature are added in parentheses) [25] (Figure 7d). The parameters of other adsorbents involved in the calculation of the RRS are shown in (Table S4). The calculated RRS of CALF‐20M‐w exceeds that of UiO‐66 and approaches ZIF‐8. Notably, UiO‐66 and ZIF‐8 have previously demonstrated radiation stability at maximum doses of 1250 kGy and 1750 kGy, respectively. Thus, it can be inferred that CALF‐20M‐w also exhibits exceptional stability against such high radiation doses, fulfilling the stringent stability requirements for adsorbents in industrial separation of radioactive Kr‐Xe mixtures.

## 3 Conclusions

In summary, we have evaluated the performance of the radiation‐stable MOF material CALF‐20M‐w for Kr/Xe molecular sieving separation under industrial PSA pressure, enabling efficient production of high‐purity Xe. The exceptional Kr selectivity arises from its precisely engineered pore‐limiting diameter (3.7 Å), achieved through structural contraction of conventional channels (3.1 Å) via periodically aligned methyl groups, which creates unidirectional molecular sieving. IAST‐predicted Kr/Xe adsorption selectivity reaches 4424 at 195 K and 5 bar, demonstrating remarkable potential for ^85^Kr removal from high‐pressure industrial Kr/Xe gas mixtures. Notably, we present the first systematic evaluation of CALF‐20M‐w's radiation stability under β‐electron beam irradiation and γ‐irradiation up to 240 kGy cumulative dose. Post‐irradiation analysis revealed no degradation in CO_2_ adsorption capacity, confirming exceptional structural integrity under ionizing radiation conditions. Complementarily, computational analyses including Crystal Orbital Overlap Population (COOP) calculations elucidate the correlation between radiation stability and chemical bond strength in CALF‐20M‐w. Furthermore, dynamic breakthrough experiments demonstrate that CALF‐20M‐w effectively captures Kr, enabling the production of high‐purity Xe (>99.9%) within defined operational cycles. These findings establish CALF‐20M‐w as a critical adsorbent for radioactive ^85^Kr removal and ultrahigh‐purity Xe production in high‐pressure Kr‐Xe mixtures, demonstrating exceptional potential for large‐scale industrial implementation.

## Conflicts of Interest

The authors declare no conflicts of interest.

## Supporting information




**Supporting File**: advs75520‐sup‐0001‐SuppMat.docx.

## Data Availability

The data that support the findings of this study are available from the corresponding author upon reasonable request.
