# Supplementary material for: Radiation‐Resistant Kr‐Selective MOF With Record Kr/Xe Selectivity at Elevated Pressure
Source: Adv Sci (Weinh). 2026 May 4;13(41):e00073. doi: 10.1002/advs.202600073 (PMC13335603; doi:10.1002/advs.202600073)
Supplement: Supplementary file 1 — Supporting File: advs75520‐sup‐0001‐SuppMat.docx. [file ADVS-13-e00073-s001.docx]

**Supporting Information**

**Radiation-Resistant Kr-Selective MOF with Record Kr/Xe Selectivity at Elevated Pressure**

**Qihang Tian, Yinhui Li, Qingkuan Meng, Shizhen Liu, Yongzheng Wang,** **Bin Chen and Heping Ma***

**Materials and Methods:**

**1.1 Materials**

All chemicals were sourced from commercial vendors and utilized without further modification.

**1.2 Synthesis of Calf-20M-w**

The synthesis of Calf-20M-w was similar to that previously reported with slight modifications.^[1]^ 3-methyl-1H-1,2,4-triazole (18 mmol, 1500 mg) and zinc oxalate (6.45 mmol, 990 mg) were added to 100 mL of deionized water in a 500 m L high pressure reactor. The resulting mixture was stirred vigorously for 2 hours and then incubated at 453 K for 3.5 days. After cooling to room temperature, the resulting precipitate was collected by filtration, further washed with methanol 3 times and dried in air to obtain Calf-20M-w.

**1.3 Characterization and measurement**

Powder X-ray diffraction (PXRD) measurements were recorded on a Bruker D8 ADVANCE diffractometer with Cu-K a radiation operating at a voltage of 50 kV and a current of 200 mA. Scanning electronic microscopy (SEM) images were recorded by a MAIA3LMH scanning electron microscope. Xenon, Krypton and carbon dioxide adsorption isotherm were obtained by BSD-PM and BSD-660 gas adsorption Analyzer from Beishide Co, Ltd. The isotherms of gases such as Xe, Kr, and CO_2_ at different temperatures and the time-varying dynamic adsorption curves adsorption isotherm were obtained using BSD-PMC gas adsorption Analyzer from Beishide Co, Ltd. Breakthrough measurements were performed using BSD-MAB Analyzer from Beishide Co, Ltd.

**1.4 Computational method**

Using the Forcite module within the Materials Studio software, we performed annealing simulations on the pristine CIF file to refine the crystal structure. The results revealed that Calf-20M-w exhibits two distinct pore diameters: 0.31 nm and 0.37 nm. The latter diameter lies between the atomic diameters of Kr and Xe, indicating that the structure enables molecular screening of Kr/Xe at the microscopic level by selectively permitting Kr transport through the pores while excluding Xe. Subsequently, the Sorption module was employed to simulate the adsorption isotherms and average loading capacities for Kr and Xe at various temperatures and pressures, providing theoretical support for the experimental observations. The simulation results demonstrate that CAF-20M-W possesses significantly greater Kr adsorption capacity compared to Xe at 195 K. This finding is consistent with experimental findings, further validating the material's superior Kr/Xe selectivity. Throughout these simulations, we utilized the cvff force field, the NVT ensemble, and Charge using QEq method. Both Electrostatic and van der Waals interactions were calculated using the Atom based summation method.

**1.5 Calculation of relative radiation stability**^[2]^

Relative radiation stability (RRS), which comprehends cross-section and coordination number of different atom and coordination bond strength, could be defined as follow:


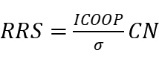
.

Where *RRS* is the relative radiation stability of coordination bond, *ICOOP* is the bond strength of coordination bond, *σ* is the absorption cross section of gamma ray at 1.17 MeV for metal node, *CN* is the coordination number of metal node.

**1.6 IAST calculation**^[3]^

IAST has been used to predict binary gas separation performance. By fitting the pure gas adsorption isotherms of gas components, the IAST selectivity can be calculated according to the equation defined as follow:


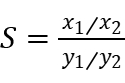


Where
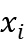
 is the molar fraction of gas 1 and gas 2 in the adsorbed phase and
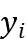
 is the molar fraction of gas 1 and gas 2 in the bulk phase.

**1.7 Pressure swing adsorption simulation**^[4]^

Model details:

The following assumptions are used to simplify the calculations:

- Material and momentum balance

1. The thermodynamic model follows the Peng-Robinson equation;
2. The momentum balance and pressure drop are described using the Ergun equation;
3. The gas accumulation in the inter-particle void space and adsorption flux to the adsorbent surface are estimated using gas-phase convection with axial dispersion; constant axial dispersion is described using a fixed axial dispersion coefficient (2.0 x 10^-5^ m^2^·s^-1^).

- Kinetic model

The flux between adsorbed molecules is calculated using the linear driving force (LDF) model with constant mass transfer coefficients.

- Equilibrium capacity model

Langmuir-Freundlich isotherms are used to describe competitive adsorption for the binary gas mixture.

- Energy balance

Isothermal model is used to describe energy balance. The gas temperature *T* and the absorbsent temperature *T_s_* are held constant and equal.

Table S1 Governing equations used to describe the material, momentum and energy balance in the adsorption column in this model.

|  | **Description** | **Equation** |
| --- | --- | --- |
| **Momentum balance** | Ergun equation | 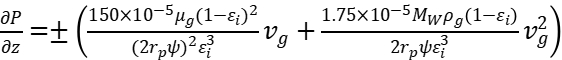 |
| **Material balance** | Plug flow with axial dispersion | 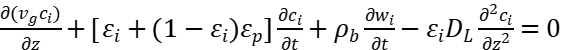 |
| **Axial dispersion** | Fixed dispersion coefficient | 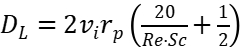 |
| **Kinetic model** | LDF model with constant MTC | 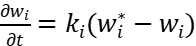 |
| **Isotherm model** | Langmuir-Freundlich isotherm | 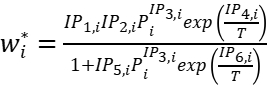 |
| **Energy balance** |  |  |
|  | Isothermal | T_i_=T_i,s_ |
|  |  |  |
| **Equation of state** | Peng-Robbinson equation | 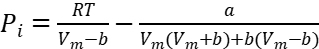 |

**Table S1: The detailed descriptions of the integrator configuration in the model**

| **Integration method** | Implicit Euler | An algorithm that performs numerical integration by evaluating the state of the system at the current time and uses this in conjunction with time derivatives to assess the system's state at the next time step. |
| --- | --- | --- |
| **Step size** | Variable step size from 10^-5^ s to 5 s | Selected to ensure fast calculation speed while maintaining calculation accuracy. |
| **Error tolerance** | 10^-5^ for absolute and relative error tolerances | Used to determine convergence by comparing the maximum change in a variable, or the numerical error in an equation, with the specified tolerances. |
| **Linear solver** | MA48 | Gaussian elimination was applied to solve a sparse, asymmetric system of *m* linear equations in *n* unknowns. |
| **Nonlinear solver** | Mixed Newton | The Newton method was used for initialization and steady-state steps, and the Fast Newton method for dynamic steps offering a balance between solution speed and robustness. |

**Notation**

| ***C_pai_*** | specific heat capacity of the adsorbed gas | ***r*** | radial coordinate of the adsorbent |
| --- | --- | --- | --- |
| ***C_ps_*** | specific heat capacity of the adsorbent | ***r_p_*** | particle radius |
| ***C_pw_*** | specific heat capacity of the wall | ***Sc*** | Schmidt number |
| ***C_vg_*** | specific gas phase heat capacity at constant volume | ***T*** | gas temperature |
| ***c_i_*** | molar fraction of component *i* | ***T_env_*** | environmental temperature |
| ***D_B_*** | internal diameter of column | ***T_s_*** | adsorbent temperature |
| ***D_L_*** | dispersion coefficient | ***T_w_*** | column wall temperature |
| ***h_b_*** | heat transfer coefficient between column and ambient | ***v_i_*** | interstitial gas velocity |
| ***h_gs_*** | heat transfer coefficient between gas and solid | ***v_g_*** | superficial gas velocity |
| ***h_w_*** | heat transfer coefficient between gas and wall | ***W_r_*** | wall thickness |
| ***IP_Xi_*** | isotherm parameter *X* for component *i* | ***w_i_*** | adsorbent loading of component *i* per unit mass of adsorbent |
| ***k_i_*** | constant mass transfer coefficient of component *i* | ***w_i_^*^*** | adsorbent loading of component *i* per unit mass of adsorbent in equilibrium with its partial pressure in gas phase |
| ***k_g_*** | heat conductivity of gas | ***z*** | axial distance coordinate |
| ***k_s_*** | heat conductivity of adsorbent | ***α_p_*** | specific particle surface area per unit length of bed |
| ***k_w_*** | heat conductivity of wall | ***ΔH_i_*** | enthalpy of adsorption for component *i* |
| ***M_W_*** | molecular weight of gas mixture | ***ε_i_*** | bed voidage |
| ***P*** | gas pressure | ***ε_p_*** | particle voidage |
| ***P_H_*** | high pressure | ***µ_g_*** | viscosity of gas mixture |
| ***P_L_*** | low pressure | ***ρ_b_*** | bulk density of adsorbent |
| ***­p­_i_*** | partial pressure of component *i* | ***ρ_g_*** | gas molar density |
| **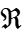** | universal gas constant | ***ρ_w_*** | column wall density |
| ***Re*** | Reynolds number | ***ψ*** | particle shape factor |

**Bed parameters and data source type:**

| **Bed parameters** | | **Data source type** |
| --- | --- | --- |
| Height of packing (m) | 1 | Designed |
| Diameter of adsorbent layer (m) | 0.2 | Designed |
| Inter-particle voidage | 0.48 | Experimental measurement |
| Intra-particle voidage | 0.7006 | Experimental measurement |
| Bulk density of adsorbent (kg *m^-3^) | 336.57 | Experimental measurement |
| Adsorbent particle radius (mm) | 1.00E-^03^ | Experimental measurement |
| Adsorbent sphericity | 1 | Estimated |

**1.8 Electron beam irradiation:**^[5]^

The electron beam irradiation (EBI) was conducted by the equipment as seen in Figure S12. First, the samples were put into the chamber on the platform and then run mechanical pumps and molecular pumps. When the pressure in the chamber was lower than 5×10^-4^ Pa, turned on the electron gun and set the beam current density and energy were 5×10^-4^ A/m^2^ and 20 keV. After that, removed the shield plate and ten min later, moved back it and the EBI process was completed.

**Beta-irradiation dose rate estimation:**^[6,7]^

1.Total power calculation:

$$Ee=20 keV=3.204\times10-15$$

$$n=\frac{J}{e}=\frac{5\times{10}^{-4}}{1.602\times{10}^{-19}}$$

$$P_{\text{ }}=n\cdot E_{e}=3.12\times{10}^{15}\times3.204\times{10}^{-15}$$

2. mass per unit area m per unit area:

$$m_{per unit area}=0.5kg/m^{2}$$

3. Dose rate:

$$Dose rate=\frac{P}{m_{per unit area}}=\frac{3.12\times{10}^{15}\times3.204\times{10}^{-15}}{0.5kg/m^{2}}=72kGy/h$$

Where: $E_{e}$, Single electron energy

$n$, Electron flux

$J$, Current density

$P_{\text{ }},$Power density

**1.9 Gamma Irradiation Measurements:**

Gamma irradiation experiments were carried out using the gamma irradiation facility, housed by Northwest Institute of Nuclear Technology, with a dose rate of 5 kGy/h, which was used to study the effects of gamma irradiation on the target sample. The dose rate was estimated based on the exposure time and distance of samples from the source and determined using Fricke dosimetry. About 200 mg activated sample was placed into a glass vial (1 cm diameter by 2 cm high). The radiation duration was 12h, 24h and 48h with total doses of 60kGy,120kGy and 240kGy respectively. The tests were carried out at ambient temperature and humidity.

**1.10 Breakthrough experiments:**

All breakthrough experiments were performed in a dynamic gas breakthrough

apparatus and were carried out using quartz columns (0.6 cm ID × 9.50 cm) artificially

filled with Calf-20M-w (917.3 mg). Powdered solid samples in the columns were activated with

helium (10 mL min^-1^) for 8 h at 413 K.


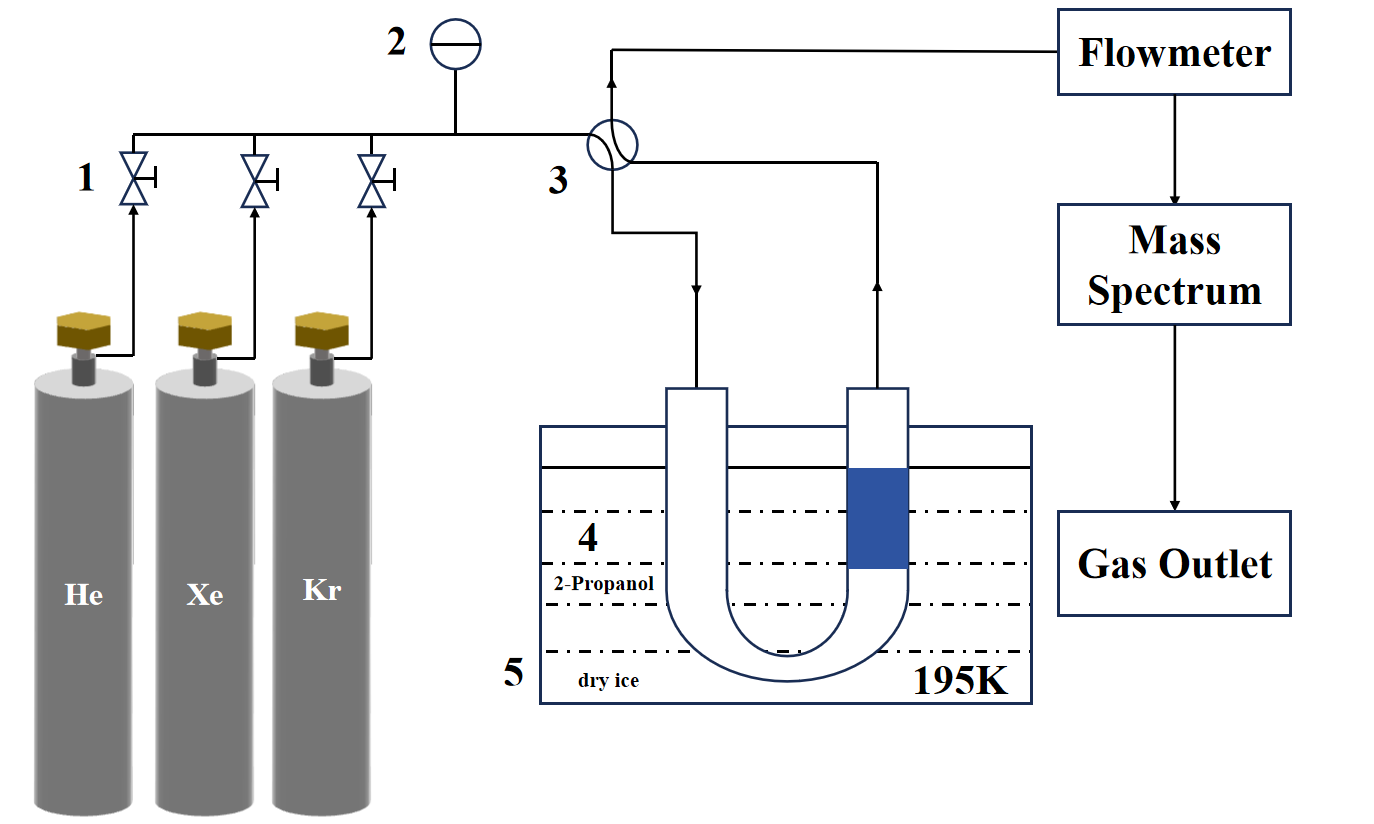


**Figure S14.** Breakthrough experiment apparatus. I: Mass flow controller, II: Pressure gauge, III: Rotary valve, IV: Heat controller, V: Sample

**Results and Discussion**


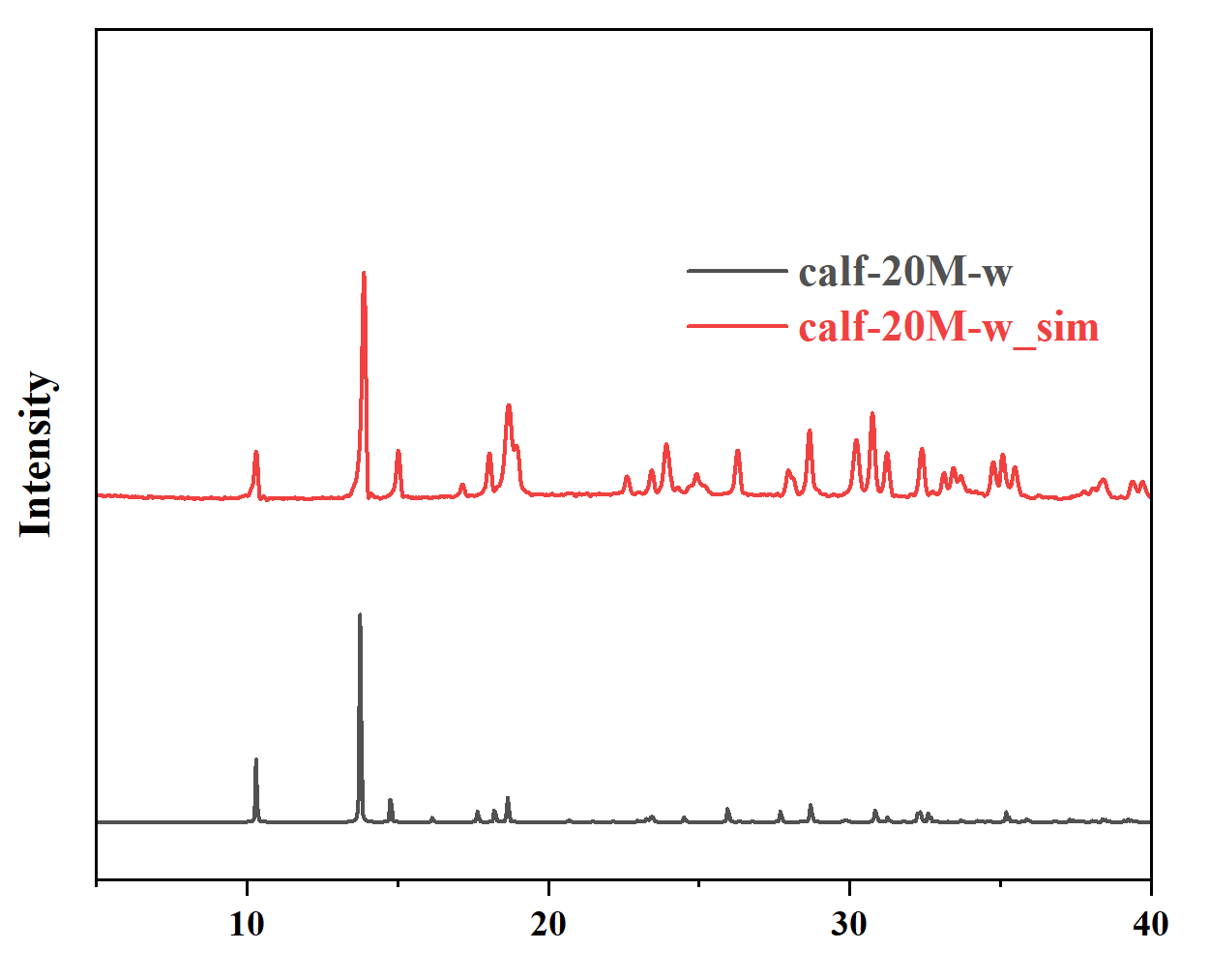


**Figure S1.** Powder X-ray diffraction (PXRD) patterns calculated from single crystal X-ray structure data (black) and experimental (red) of Calf-20M-w.


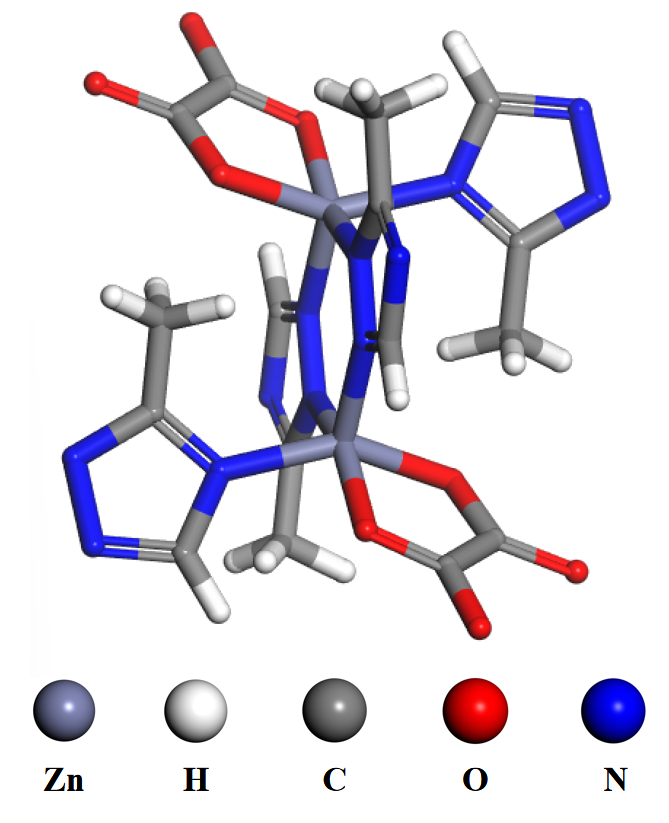


**Figure S2.** Coordination of Zn in Calf-20M-w


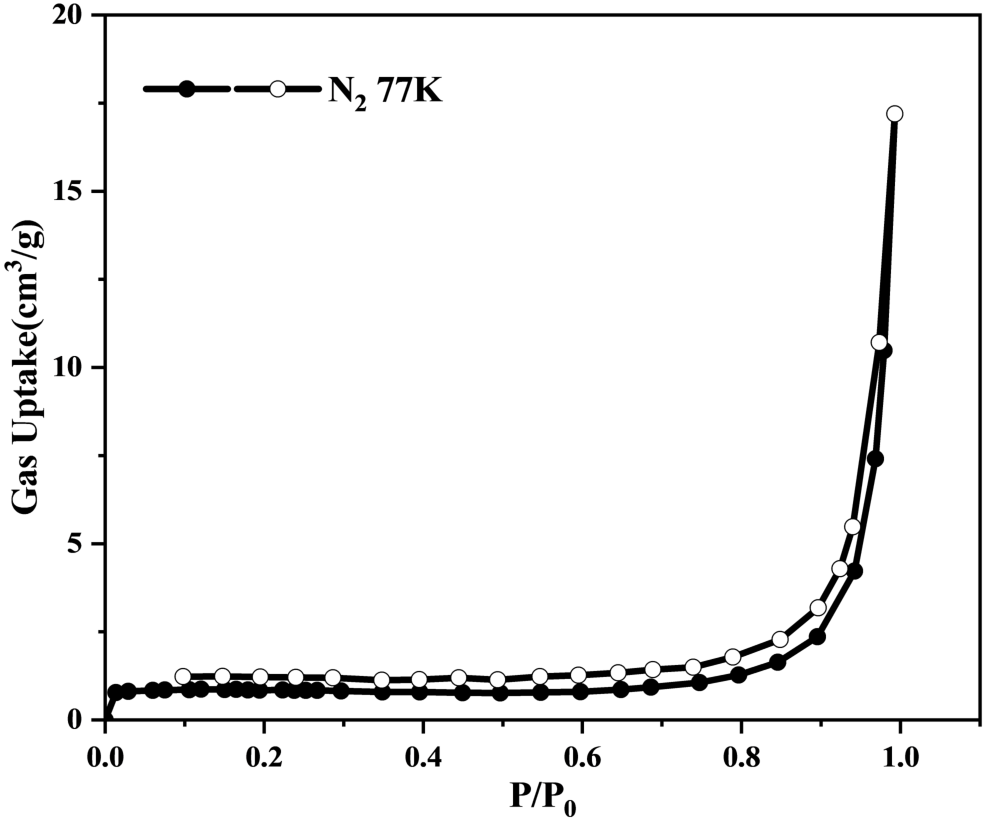


**Figure S3.** N_2_ adsorption isotherms for Calf-20M-w at 77 K.


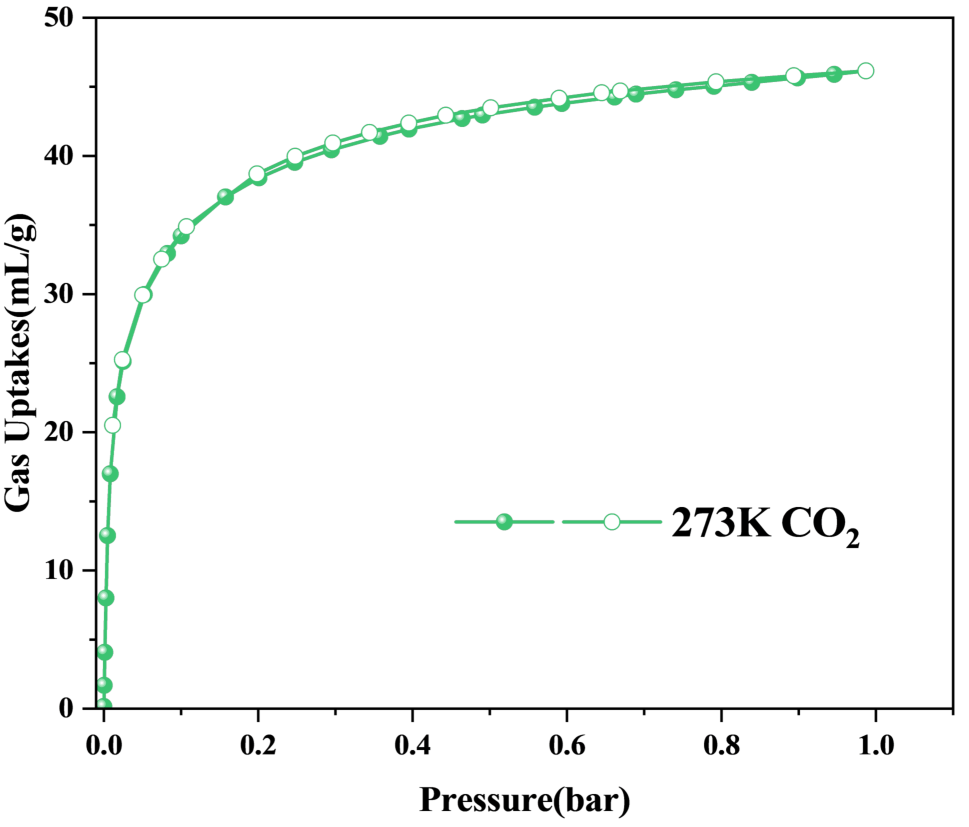


**Figure S4.** CO_2_ adsorption isotherms for Calf-20M-w at 273 K.


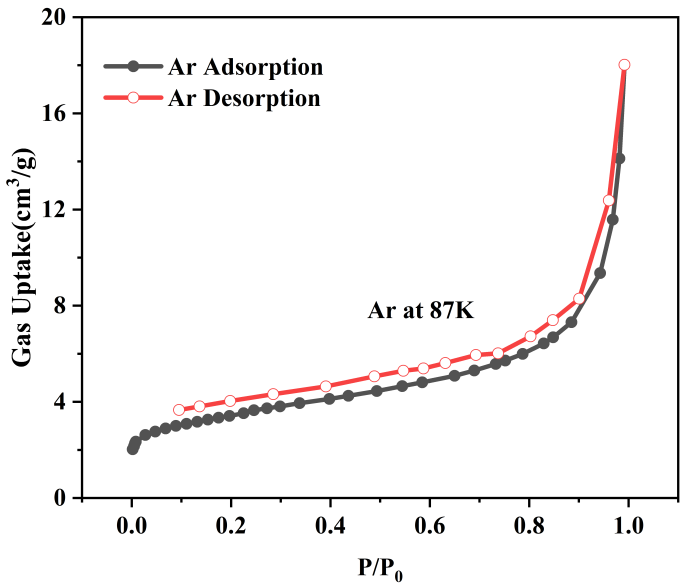


**Figure S5.** The Ar adsorption and desorption isotherms of Calf-20M-w at 87 K.


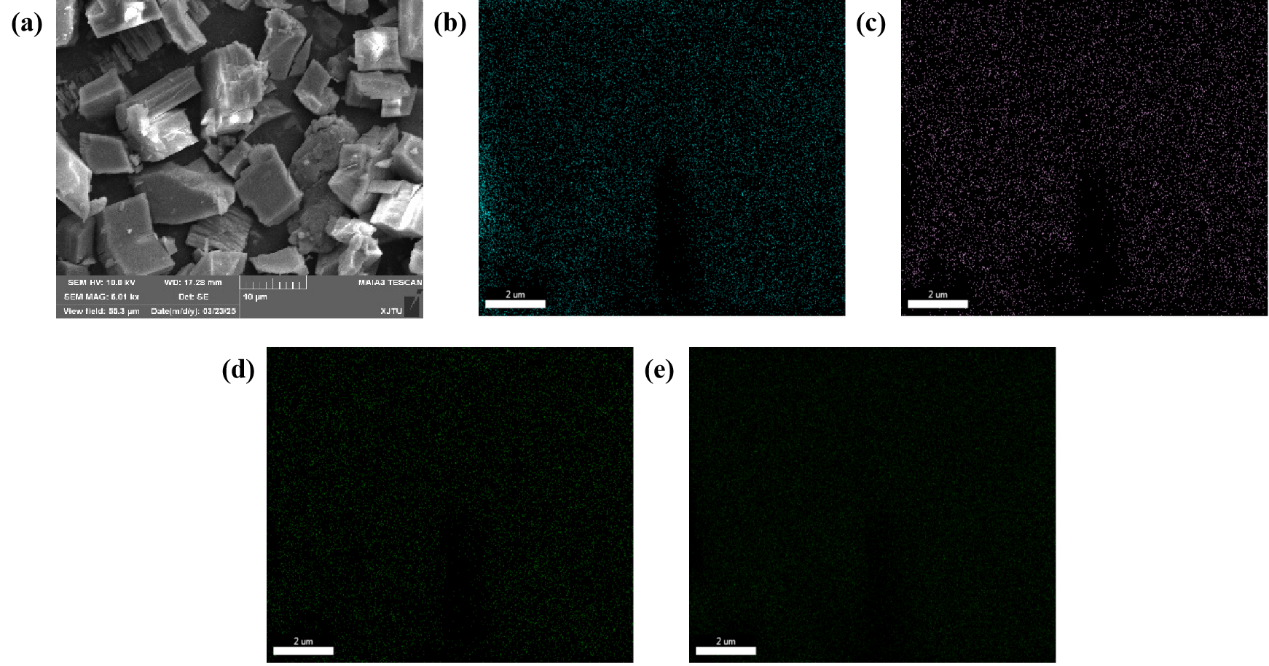


**Figure S6.** (a) The SEM images of Calf-20M-w; (b-e) The elemental distribution of C, N, O, and Zn.


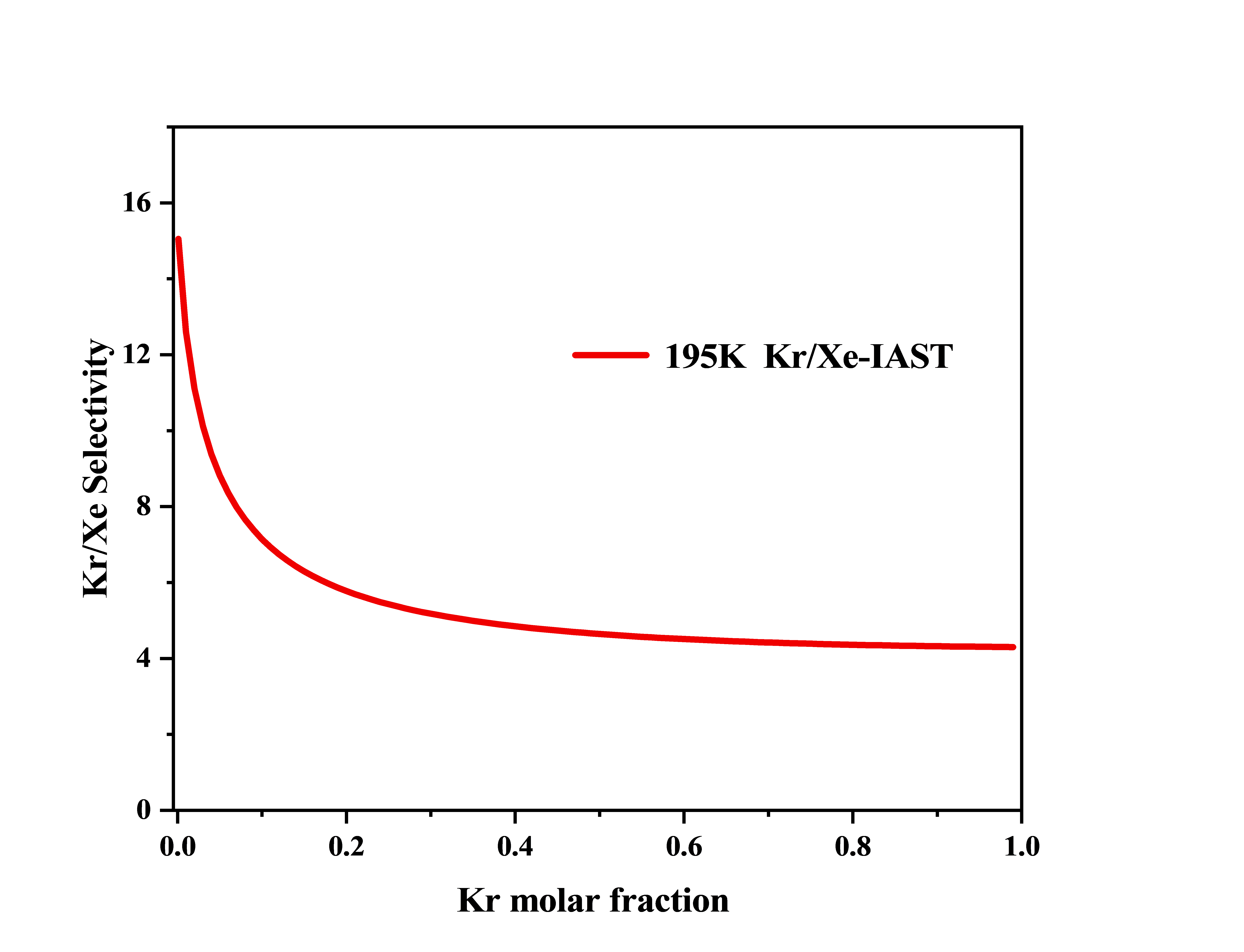


**Figure S7.** Plot of IAST selectivity of Kr/Xe as a function of Kr fraction at 1 bar and 195 K.


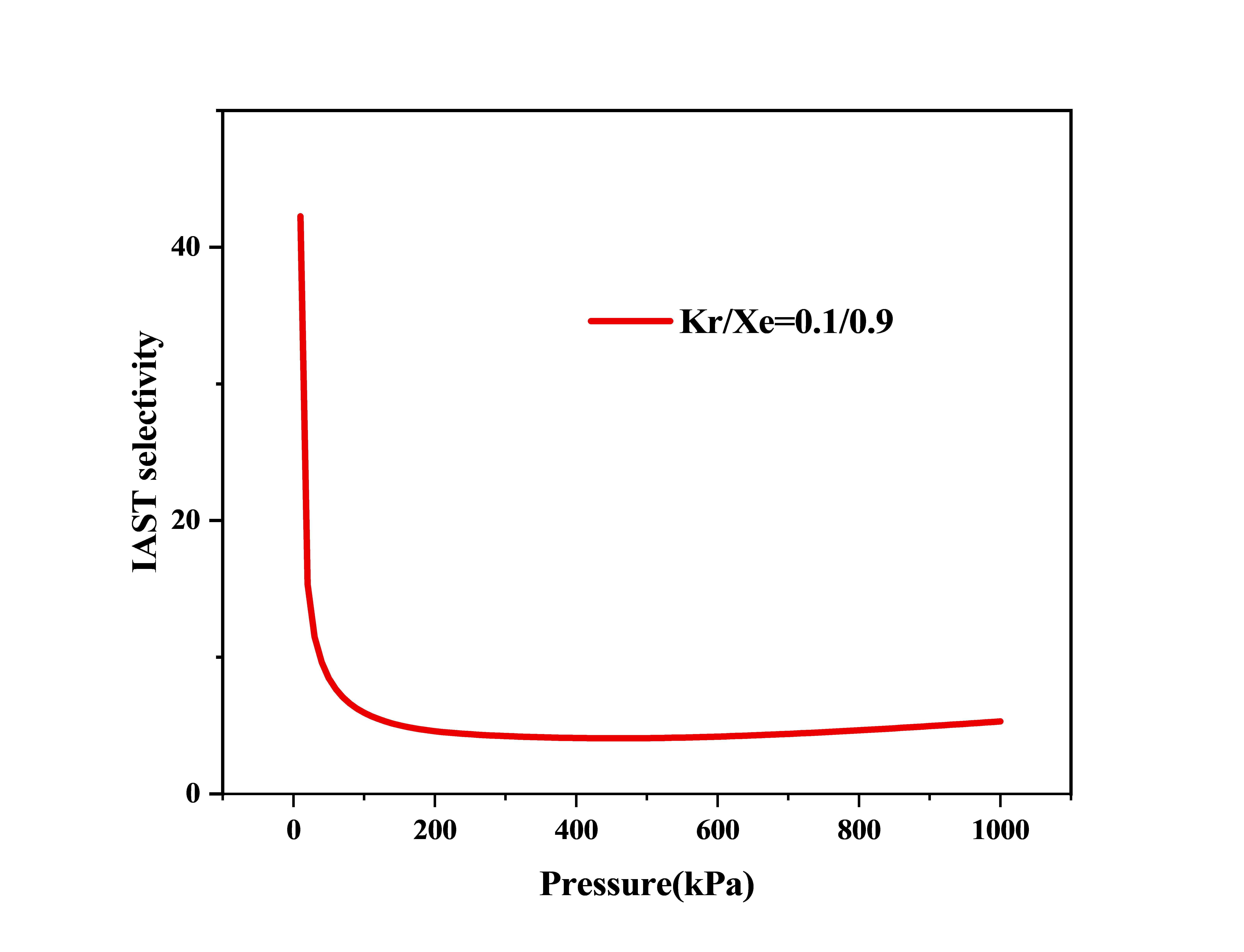


**Figure S8.** IAST selectivity of Kr/Xe at (0.1/0.9 v/v) at 224 K and 10 bar


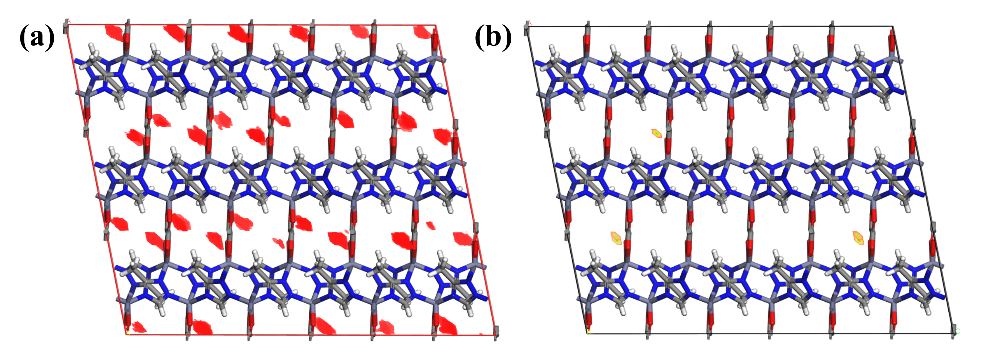


**Figure S9.** Adsorption images of (a) Kr and (b) Xe in Calf-20M-w obtained from GCMC simulations.


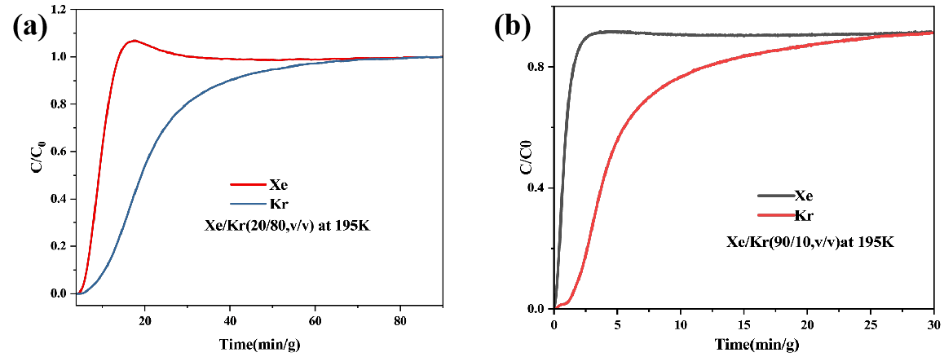


**Figure S10.** Breakthrough experiments. (a) 195K under Kr/Xe (20:80, v/v) with Calf-20M-w (b) 195K under Kr/Xe (90:10, v/v) with Calf-20M-w


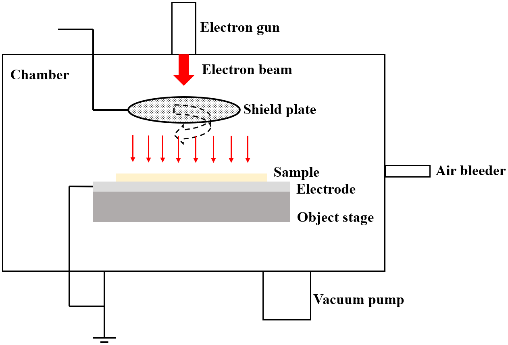


**Figure S11.**The electron beam irradiation (EBI) equipment.


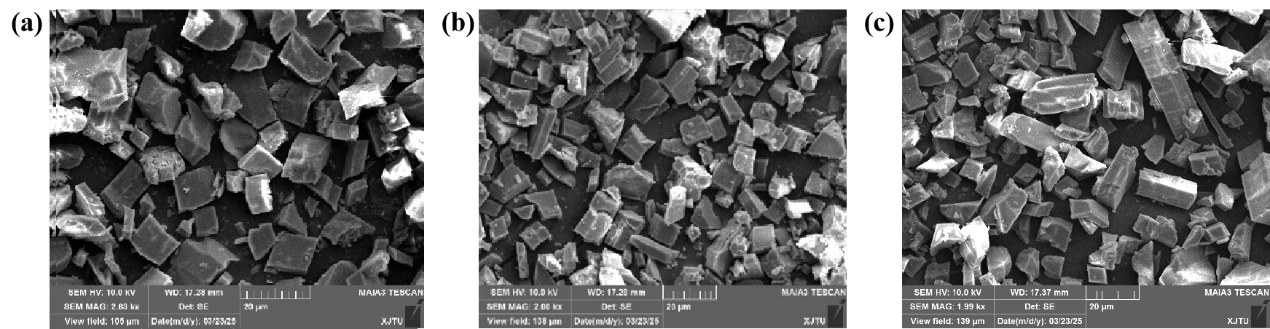


**Figure S12.** SEM images of (a) Calf-20M-w before treatment of radiation, (b-c) Calf-20M-w after treatment of β and γ radiation.

**Table S2:** Elemental analysis of Calf-20M-w measured with EDX and theoretically calculated

| **Element** | **Theoretical element content (atomic %)** | **Experimental element content (atomic %)** |
| --- | --- | --- |
| C | 40% | 43.24% |
| N | 30% | 31.13% |
| O | 20% | 15.15% |
| Zn | 10% | 10.47% |

**Table S3:** The selectivity of the Xe / Kr separation of the benchmark porous material.^[8]^

| **Kr Selective Sorbent** | **20:80 Xe/Kr Selectivity** | **Xe Selective Sorbent** | **20:80 Xe/Kr Selectivity** |
| --- | --- | --- | --- |
| Calf-20M-w | 4424 (This work) | Co_3_(C_4_O_4_)_2_(OH)_2_^[9]^ | 69.7^[b]^ |
| Mn(ina)_2_^[10]^ | 170^[b]^ | MOF-74-Ni^[11]^ | 4^[a]^ |
| C-Suc-750^[12]^ | 105^[b][c]^ | MOF-74-Co^[11]^ | 3.91^[c]^ |
| FS-CuBTC_UM_^[13]^ | 20^[b]^ | MOF-74-Mg^[11]^ | 3.76^[c]^ |
|  |  | Ag@MOF-74Ni^[14]^ | 11.5^[b]^ |
|  |  | HKUST-1^[11]^ | 2.6^[a]^ |
|  |  | Co_3_(HCOO)_6_^[15]^ | 11^[b]^ |
|  |  | SBMOF-2 | 10^[b]^ |
|  |  | CC3^[16]^ | 12.5^[b]^ |
|  |  | CROFOUR-1-Ni | 22^[a]^ |
|  |  | CROFOUR-2-Ni | 15.5^[a]^ |

[a] From breakthrough experiment. [b] From IAST calculation. [c] Henry’s constant based on single component isotherm.

**Table S4:** Relative radiation stability for Calf-20M-w as compared to benchmark radiation stable MOFs.

| **Materials** | **Chemical bond** | **ICOOP of chemical bond** | **Cross-section of metal node at 1.17 MeV** | **Coordination number of metal node** | **RRS** | **Irradiation strength (kGy)** |
| --- | --- | --- | --- | --- | --- | --- |
| Calf-20M-w | Zn-O | 0.134 | 5.97 | 5 | 0.113 | 240 |
| TIFSIX-3-Co | Co-F | 0.058 | 5.33 | 2 | 0.022 | 50 |
| NbOFFIVE-1-Ni | Nb-O | 0.142 | 8.31 | 2 | 0.034 | 50 |
| ZJU-74-Pd | Co-N | 0.137 | 5.33 | 2 | 0.051 | 1000 |
| UiO-66(Zr) | Zr-O | 0.108 | 8.12 | 4 | 0.053 | 1250 |
| MOF-11 | Cu-O | 0.169 | 5.76 | 4 | 0.117 | 20 |
| ZIF-8 | Zn-N | 0.202 | 5.97 | 4 | 0.136 | 1750 |
| MIL-100(Al) | Al-O | 0.169 | 2.55 | 4 | 0.264 | 1750 |

**Table S5:** The 2-bed PSA simulation results of Calf-20m-w.

| **Adsorbent** | **Calf-20M-w** |
| --- | --- |
| **Purity of Xe** **at column-top (%)** | 99 |
| **Production efficiency of Xe (mol kg^-1^ h^-1^)** | 2.83 |
| **Total energy consumption (MJ kg (Xe)^-1^)** | 21.69 |

**Table S6:** The binding energy difference between Calf-20M-w.

| **Gas** | **Binding energy(kJ/mol)** |
| --- | --- |
| Xe | -28.67 |
| Kr | -24.64 |

[1] X. Wang, M. Alzayer, A. J. Shih, S. Bose, H. Xie, S. M. Vornholt, C. D. Malliakas, H. Alhashem, F. Joodaki, S. Marzouk, G. Xiong, M. Del Campo, P. Le Magueres, F. Formalik, D. Sengupta, K. B. Idrees, K. Ma, Y. Chen, K. O. Kirlikovali, T. Islamoglu, K. W. Chapman, R. Q. Snurr, O. K. Farha, *J. Am. Chem. Soc.* **2024**, *146*, 3943.

[2] B. Chen, S. Liu, Y. Wang, T. Cao, L. Chen, L. Miao, S. Wu, H. Ma, *Adv Funct Materials* **2025**, 2420105.

[3] C. M. Simon, B. Smit, M. Haranczyk, *Computer Physics Communications* **2016**, *200*, 364.

[4] J. Cui, Z. Zhang, L. Yang, J. Hu, A. Jin, Z. Yang, Y. Zhao, B. Meng, Y. Zhou, J. Wang, Y. Su, J. Wang, X. Cui, H. Xing, *Science* **2024**, *383*, 179.

[5] M. Li, H. Niu, K. Shang, J. Lang, Y. Gao, B. Li, J. Zhao, Z. Li, Y. Feng, S. Li, *High Voltage* **2024**, *9*, 528.

[6] S. K. Elsaidi, M. H. Mohamed, A. S. Helal, M. Galanek, T. Pham, S. Suepaul, B. Space, D. Hopkinson, P. K. Thallapally, J. Li, *Nat Commun* **2020**, *11*, 3103.

[7] A. M. Hastings, M. Fairley, M. C. Wasson, D. Campisi, A. Sarkar, Z. C. Emory, K. Brunson, D. B. Fast, T. Islamoglu, M. Nyman, P. C. Burns, L. Gagliardi, O. K. Farha, A. E. Hixon, J. A. LaVerne, *Chem. Mater.* **2022**, *34*, 8403.

[8] M. H. Mohamed, S. K. Elsaidi, T. Pham, K. A. Forrest, H. T. Schaef, A. Hogan, L. Wojtas, W. Xu, B. Space, M. J. Zaworotko, P. K. Thallapally, *Angew Chem Int Ed* **2016**, *55*, 8285.

[9] L. Li, L. Guo, Z. Zhang, Q. Yang, Y. Yang, Z. Bao, Q. Ren, J. Li, *J. Am. Chem. Soc.* **2019**, *141*, 9358.

[10] H. Wang, M. Warren, J. Jagiello, S. Jensen, S. K. Ghose, K. Tan, L. Yu, T. J. Emge, T. Thonhauser, J. Li, *J. Am. Chem. Soc.* **2020**, *142*, 20088.

[11] J. J. I. Perry, S. L. Teich-McGoldrick, S. T. Meek, J. A. Greathouse, M. Haranczyk, M. D. Allendorf, *J. Phys. Chem. C* **2014**, *118*, 11685.

[12] F. Chen, F. Zheng, X. Huang, Z. Chu, H. Sun, L. Yang, Q. Yang, Z. Zhang, Q. Ren, Z. Bao, *Advanced Materials* **2024**, 2409474.

[13] M. H. Mohamed, I. Elzeny, J. Samuel, Y. Huang, A. S. Helal, M. Galanek, W. Xu, S. Y. Kim, T. Pham, L. Miller, A. Hogan, B. Space, J. Li, S. K. Elsaidi, *ACS Appl. Mater. Interfaces* **2024**, *16*, 29364.

[14] J. Liu, D. M. Strachan, P. K. Thallapally, *Chem. Commun.* **2013**, *50*, 466.

[15] H. Wang, K. Yao, Z. Zhang, J. Jagiello, Q. Gong, Y. Han, J. Li, *Chem. Sci.* **2013**, *5*, 620.

[16] L. Chen, P. S. Reiss, S. Y. Chong, D. Holden, K. E. Jelfs, T. Hasell, M. A. Little, A. Kewley, M. E. Briggs, A. Stephenson, K. M. Thomas, J. A. Armstrong, J. Bell, J. Busto, R. Noel, J. Liu, D. M. Strachan, P. K. Thallapally, A. I. Cooper, *Nature Mater* **2014**, *13*, 954.
